# Surface roughness of contemporary resin composites after exposure to energy drinks

**DOI:** 10.2340/biid.v13.46583

**Published:** 2026-08-04

**Authors:** Jhonatan Vilca-Mayta, José Giancarlo Tozo-Burgos, Marco Sánchez-Tito

**Affiliations:** a School of Dentistry, Faculty of Health Sciences, Universidad Privada de Tacna, Tacna, Peru; b Research Group on Dental Biomaterials and Natural Products, Faculty of Health Sciences, Universidad Privada de Tacna, Tacna, Peru

**Keywords:** Energy drinks, resin composites, surface roughness, erosive degradation, citric acid, restorative materials

## Abstract

**Objective:**

This study aimed to evaluate the effect of three energy drinks (Red Bull^®^, Volt^®^, and 360 Energy Drink^®^) on the surface roughness of three contemporary resin composites: Filtek Z350 XT, PALFIQUE LX5, and Vittra APS.

**Materials and methods:**

An *in vitro* experimental design was conducted with 108 resin specimens (*n* = 12 per group). After polishing, baseline roughness (Ra, µm) was measured using a profilometer. Specimens were immersed in one of the three beverages for 1 and 7 days, with daily renewal. Roughness was recorded at T₀, T₁, and T₂. Data were analyzed using a linear mixed-effects model with Sidak-adjusted post hoc comparisons (α = 0.05).

**Results:**

All resins composites exhibited a progressive increase in Ra from T₀ to T₂. Filtek Z350 XT showed the greatest resistance to surface degradation, while PALFIQUE LX5 and Vittra APS were more susceptible. Red Bull^®^ and 360 Energy Drink^®^ induced greater roughness increases than Volt^®^ (*p* < 0.05). Although most roughness values remained at or near the clinically accepted threshold of Ra ≤ 0.2 µm, a slight increase above this threshold was observed for PALFIQUE LX5 exposed to 360 Energy Drink^®^ at T₂. A significant resin × time interaction (*p* = 0.034) indicated that degradation patterns depended on composite type.

**Conclusion:**

Energy drinks promoted measurable increases in the surface roughness of all evaluated resin composites. Filtek Z350 XT showed greater resistance to roughness changes, whereas PALFIQUE LX5 and Vittra APS demonstrated higher susceptibility to acidic degradation.

KEY MESSAGES

1. Energy drinks promoted progressive surface roughness changes in all evaluated resin composites, with degradation patterns varying according to both beverage and material composition.
2. Filtek Z350 XT demonstrated greater resistance to roughness changes, whereas PALFIQUE LX5 and Vittra APS showed higher susceptibility after exposure to energy drinks.

## Introduction

Resin composites are widely used in restorative dentistry because of their favorable esthetic and functional properties. However, their long-term clinical performance may be compromised by exposure to the oral environment and dietary habits, particularly the frequent consumption of acidic beverages [1, 2].

Energy drink consumption has increased markedly, especially among adolescents and young adults [3, 4]. These beverages commonly contain caffeine, taurine, sugars, vitamins, and acidic components that may adversely affect oral tissues and restorative materials [5]. Regular intake has also been associated with several adverse health effects [6–9]. Their low pH has been associated with erosion of dental hard tissues [10] and with changes in the surface properties of restorative materials.

Several *in vitro* studies have shown that exposure to energy drinks increases surface roughness and alters the morphology of resin composites [11–13]. Kose et al. reported significant roughness increases in Charisma Diamond One after 7 days of immersion in Monster^®^ and Burn^®^ energy drinks [11]. Similarly, Al-Samadani observed progressive roughness increases in Filtek Z350 XT after prolonged immersion in Red Bull^®^, Bison™, and Power Horse™, with Red Bull^®^ producing the greatest effect [12]. Kolarovszki et al. further showed that composites with irregular filler distribution were more susceptible to surface degradation, whereas materials with more uniform particles showed greater resistance [13].

Despite advances in composites technology, limitations such as polymerization shrinkage, wear resistance, postoperative sensitivity, and concerns regarding long-term clinical performance remain relevant [14]. Among the surface properties of composites, roughness and microhardness are particularly important because increased roughness favors bacterial adhesion, plaque accumulation, extrinsic staining, and material deterioration, whereas reduced hardness may decrease resistance to wear and scratching [15]. Surface topography is also influenced by composite type and by finishing and polishing procedures, which can directly affect bacterial adhesion [16]. Therefore, evaluating surface roughness after acidic exposure is clinically relevant for understanding early degradation patterns in restorative materials.

Although previous studies have evaluated the effects of energy drinks on conventional or widely used resin composites, limited evidence is available for newer materials incorporating modified resin matrices and advanced photopolymerization systems. In particular, PALFIQUE LX5 and Vittra APS have not been extensively investigated regarding their surface stability after exposure to energy drinks. Because the erosive potential of energy drinks depends not only on pH but also on their overall formulation, this study evaluated three commercially available beverages selected to represent different physicochemical profiles, including variations in acidity and ingredient composition. Among them, 360 Energy Drink^®^ was included because, although it shares characteristics with other commercial energy drinks, its effects on dental restorative materials have not previously been investigated.

Therefore, the present study aimed to evaluate the effect of Red Bull^®^, Volt^®^, and 360 Energy Drink^®^ on the surface roughness of three contemporary resin composites: Filtek Z350 XT, PALFIQUE LX5, and Vittra APS.

## Materials and methods

### Study design, ethical aspects, and sample size calculation

**Figure 1:**
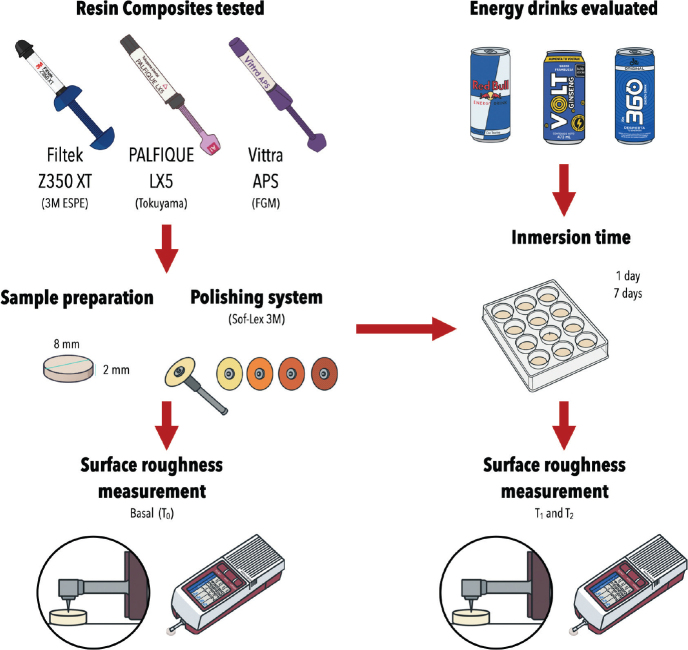
Experimental design and work flowchart for evaluating the effect of energy drinks on the surface roughness of resin composites.

This *in vitro* experimental study was approved by the Institutional Research Ethics Committee (protocol FACSA-CEI/043-05-2025). Sample size was calculated using GPower 3.1.9.7 with a fixed-effects, one-way analysis of variance (ANOVA), effect size *f* = 0.40, significance level of 5% (α = 0.05), test power of 80% (1-β = 0.8), and nine groups. A minimum of 108 specimens was required (*n* = 12 per group). Group distribution corresponded to the combination of three composites and three energy drinks. A work flowchart (Figure 1) summarizes the experimental procedures, and the composition of materials is presented in Table 1.

**Table 1:** Composition and characteristics of the materials used in the study.

Resin composites						
Resin composite	Manufacturer	Shade	Organic matrix	Inorganic filler	Filler content	Photoinitiator system
Filtek Z350 XT	3M ESPE, St. Paul, MN, USA	A1	Bis-GMA, UDMA, TEGDMA, Bis-EMA	Silica (20 nm) and zirconia (4–11 nm) nanoparticles; nano-clustered fillers (0.6–1.4 µm)	78.5 wt%/63.3 vol%	Camphorquinone (CQ)
PALFIQUE LX5	Tokuyama Dental Co., Tokyo, Japan	A1	Bis-GMA, TEGDMA, UDMA	Nano- and micro-hybrid silica–zirconia fillers	82 wt%/70 vol%	Camphorquinone (CQ) + RAD (radical-amplifying system)
Vittra APS	FGM Dental Group, Santa Catarina, Brazil	A1	Bis-GMA, UDMA, TEGDMA	Silica–zirconia nanoparticles	89 wt%/74 vol%	APS (Advanced Polymerization System), camphorquinone-free

Energy drinks						

Beverage	Manufacturer	Main ingredients		Sugar	Caffeine	pH

Red Bull^®^	Red Bull GmbH, Austria	Carbonated water, sucrose, glucose, citric acid, taurine, caffeine, inositol, B-group vitamins, artificial flavors		11 g/100 mL	32 mg/100 mL	3.3
Volt^®^	Ajegroup S.A., Peru	Carbonated water, sugar, citric acid, caffeine, taurine, inositol, vitamins B3, B5, B6, B12, artificial flavors		10 g/100 mL	32 mg/100 mL	2.8–3.0
360 Energy Drink^®^	ISM, Ayacucho, Peru	Carbonated water, sucrose, glucose, citric acid, taurine, caffeine, B-group vitamins, artificial flavors		11 g/100 mL	32 mg/100 mL	2.9–3.2

### Specimen preparation

A total of 108 disc-shaped specimens were fabricated from the composites evaluated in this study. An A1 shade from the VITA Classical shade guide (VITA Zahnfabrik, Bad Säckingen, Germany) was selected to standardize color among materials. Specimens (8 mm in diameter × 2 mm in thickness) were prepared using stainless-steel molds coated with a thin glycerin layer to facilitate removal [17]. Each composite was inserted in a single increment using a Teflon spatula, covered with a Mylar strip, and compressed with a glass slide to obtain a flat and smooth surface for roughness evaluation [11]. Both sides were light-cured for 20 s using a light-emitting diode (LED) curing unit (VALO LED; Ultradent Products, South Jordan, UT, USA; 1600 mW/cm²). Specimens were then stored in saline solution at room temperature for 24 hours. Discs presenting bubbles, edge irregularities, surface porosities, heterogeneous filler distribution, or thickness variations were excluded to ensure specimen standardization and measurement reliability [18].

### Finishing and polishing

Finishing and polishing were performed on a single side of each disc (the upper surface), which was designated as the experimental surface of the study. The Sof-Lex™ XT disc system (3M ESPE, Minnesota, USA) was used in a sequential manner, from the coarsest to the finest grit, attached to a low-speed handpiece operating at 10,000–15,000 rpm. Each disc was applied for 30 s. Between each step, specimens were rinsed with distilled water and dried with lint-free absorbent paper. Discs were replaced after every two specimens to ensure consistent abrasiveness. After polishing, all specimens were stored in saline at room temperature.

### Baseline roughness measurement (T₀)

Surface roughness (Ra) was assessed using a profilometer (Mitutoyo America Corporation, Aurora, IL, USA) with parameters of 0.4 g load, 0.8 mm cut-off length, and 0.5 mm/s speed [19]. Three readings were obtained per specimen, and the mean value was recorded [8].

### Exposure to energy drinks

Specimens were randomly allocated into nine groups (*n* = 12), according to composite and beverage type. Each group was immersed in 5 mL of Red Bull^®^ (Red Bull GmbH, Fuschl am See, Austria), Volt^®^ (Ajegroup, Lima, Peru), or 360 Energy Drink^®^ (ISM, Ayacucho, Peru).

Specimens were maintained under continuous immersion at room temperature for 7 days, following protocols previously shown to induce measurable changes in the surface properties of composites under acidic exposure conditions [11]. Beverages were renewed daily. After immersion, specimens were rinsed and stored in saline solution at room temperature. Saline was selected to maintain standardized and reproducible conditions while minimizing the chemical variability associated with artificial saliva [11].

### Roughness measurements at T₁ and T₂

After 1 (T₁) and 7 (T₂) days of immersion, specimens were rinsed with saline and dried with absorbent paper, and surface roughness was reassessed under the same conditions described for baseline.

### Statistical analysis

Data were analyzed using Stata 17.0 (StataCorp LLC, College Station, TX, USA). Given the repeated-measures design, a linear mixed model was applied to simultaneously evaluate the fixed effects of composite type, energy drink, and time while accounting for random effects associated with specimen variability. Post hoc pairwise comparisons were adjusted with the Sidak method. A significance level of 5% was adopted.

## Results

Descriptive surface roughness (Ra) values for the three composites at each time interval are presented in Table 2. Overall, all materials exhibited a progressive increase in roughness from T₀ to T₂, although the extent of change varied according to both composite type and energy drink. Vittra APS showed a marked rise when exposed to Red Bull^®^, increasing from 0.092 ± 0.040 at T₀ to 0.133 ± 0.048 at T₂, while consistently elevated values were observed across all intervals with 360 Energy Drink^®^. PALFIQUE LX5 demonstrated a substantial increase after immersion in Volt^®^ and 360 Energy Drink^®^, reaching one of the highest roughness values recorded at T₂ (0.207 ± 0.135). By contrast, Filtek Z350 XT exhibited more stable behavior, with comparatively lower mean values under most conditions.

**Table 2:** Descriptive statistics of surface roughness values (Ra, μm) for the resin composites exposed to energy drinks at the three evaluation times.

Resin composite	Energy drink	T0 (Mean ± SD)	T1 (Mean ± SD)	T2 (Mean ± SD)
Filtek Z350 XT	Red Bull	0.103 ± 0.045	0.112 ± 0.053	0.113 ± 0.055
	Volt	0.106 ± 0.062	0.092 ± 0.072	0.117 ± 0.072
	360	0.118 ± 0.095	0.113 ± 0.094	0.134 ± 0.092
Vittra	Red Bull	0.092 ± 0.040	0.125 ± 0.041	0.133 ± 0.048
	Volt	0.119 ± 0.066	0.137 ± 0.044	0.130 ± 0.086
	360	0.171 ± 0.128	0.192 ± 0.133	0.184 ± 0.130
Palfique LX5	Red Bull	0.091 ± 0.053	0.126 ± 0.073	0.125 ± 0.094
	Volt	0.116 ± 0.090	0.169 ± 0.091	0.129 ± 0.068
	360	0.113 ± 0.069	0.199 ± 0.096	0.207 ± 0.135

Linear mixed model analysis revealed a significant resin × time interaction (*p* = 0.034), indicating that changes in surface roughness over time were dependent on the type of composite. In this study, resistance to surface degradation was operationally defined as a lower magnitude of roughness increase over time, rather than absolute Ra values at a single time point. Accordingly, materials exhibiting smaller time-dependent increases in roughness were considered more resistant. No other interactions were statistically significant (*p* > 0.05). Significant main effects were found for composite (*p* < 0.001), beverage (*p* = 0.003), and time (*p* < 0.001) (Table 3).

**Table 3:** Linear mixed-effects model for surface roughness (Ra, µm).

Variation source	df (num, den)	*F*	*p*
Composite	2, 96	15.42	< 0.001*
Energy drink	2, 96	6.21	0.003*
Time	2, 192	22.85	< 0.001*
Composite × Energy drink	4, 96	1.87	0.120
Composite × Time	4, 192	2.64	0.034*
Energy drink × Time	4, 192	1.52	0.205
Composite × Energy drink × Time	8, 192	1.12	0.349

Significance level set at *p* < 0.05.

Post hoc comparisons based on estimated marginal means (Table 4) showed that Vittra APS (ΔRa = 0.021 µm; *p* = 0.001) and PALFIQUE LX5 (ΔRa = 0.018 µm; *p* = 0.003) presented significantly higher surface roughness than Filtek Z350 XT. No significant difference was observed between Vittra APS and PALFIQUE LX5 (*p* = 0.642). Regarding energy drinks, Red Bull^®^ produced significantly greater roughness than Volt^®^ (ΔRa = 0.015; *p* = 0.007) and 360 Energy Drink^®^ (ΔRa = 0.020; *p* < 0.001). No significant difference was detected between Volt^®^ and 360 Energy Drink^®^ (*p* = 0.412).

**Table 4:** Adjusted multiple comparisons between resin composites and energy drinks.

Comparison	Mean difference (ΔRa)	95% CI	Adjusted *p*-value
Filtek Z350 XT vs Vittra	0.021	0.010, 0.032	0.001*
Filtek Z350 XT vs PALFIQUE LX5	0.018	0.006, 0.029	0.003*
Vittra vs PALFIQUE LX5	–0.003	–0.014, 0.009	0.642
Red Bull vs Volt	0.015	0.004, 0.027	0.007*
Red Bull vs 360 energy drink	0.020	0.009, 0.031	< 0.001*
Volt vs 360 energy drink	0.005	–0.006, 0.016	0.412

Post hoc comparisons with Sidak adjustment based on estimated marginal means from the linear mixed-effects model. ΔRa expressed in micrometers (µm).

* Adjusted *p*-values considered significant when < 0.05. Positive ΔRa values indicate higher roughness in the second group of the comparison.

## Discussion

This study evaluated the effect of three energy drinks on the surface roughness of three resin composites at different immersion periods. Findings demonstrated that all materials exhibited progressive roughness increases after exposure, although the magnitude of change varied according to both resin type and beverage. These results support the initial hypothesis, showing that interaction between acidic energy drinks and composites induces surface deterioration reflected in higher Ra values.

Among the materials, Filtek Z350 XT showed the most stable behavior, with minimal variations across the evaluated intervals. By contrast, Vittra APS and PALFIQUE LX5 exhibited more pronounced increases in roughness, particularly following exposure to 360 Energy Drink^®^. This pattern aligns with previous reports indicating that resistance to acidic challenges depends largely on filler characteristics. Filtek Z350 XT, a nanofilled composite containing silica/zirconia nanoclusters, presents a more uniform distribution of smaller fillers, producing a compact and homogeneous surface less prone to chemical degradation [11, 13]. In contrast, composites with larger or irregularly distributed fillers are more susceptible to dissolution and surface breakdown [13].

The marked deterioration observed in PALFIQUE LX5 may be attributed to its matrix composition, which includes Bis-GMA, UDMA, and TEGDMA. TEGDMA, a low-molecular-weight monomer, is particularly vulnerable to water sorption and hydrolytic degradation [20, 21]. These features facilitate plasticization of the matrix and filler particle dislodgement under acidic conditions. Despite its high filler loading (82 wt%), the hybrid distribution of nano- and micro-fillers is less homogeneous than the nanocluster architecture of Filtek Z350 XT, allowing easier acid penetration. Consistently, Abouelmagd and Basheer reported that composites with higher TEGDMA content showed significant hardness loss and increased roughness after exposure to acidic beverages [1].

Vittra APS also exhibited marked increases in surface roughness. Although it has a high filler content (89 wt%), its fillers consist of isolated nanoparticles rather than clustered structures, which may reduce surface compaction and resistance to acidic degradation. In addition, the APS initiator system, which does not rely on camphorquinone, could influence polymerization behavior and the chemical stability of the resin matrix under acidic conditions. However, this interpretation remains speculative because the degree of conversion and chemical stability were not directly evaluated in the present study [22, 23]. These factors may partially explain the greater susceptibility of Vittra APS compared with Filtek Z350 XT, despite its higher filler loading.

The erosive effect of energy drinks was also evident. Red Bull^®^ and 360 Energy Drink^®^ produced greater roughness increases than Volt^®^, particularly in PALFIQUE LX5 and Vittra APS. Although most roughness values remained within or close to the clinically accepted threshold of Ra ≤ 0.2 µm, PALFIQUE LX5 exposed to 360 Energy Drink^®^ slightly exceeded this limit at T₂ (mean Ra = 0.207 µm). This finding suggests that prolonged acidic exposure may increase the risk of plaque retention, staining, and surface deterioration in certain resin–beverage combinations. These results are consistent with Al-Samadani, who reported progressive surface degradation of composites after prolonged exposure to energy drinks [12].

Interestingly, despite presenting the lowest pH (2.8–3.0), Volt^®^ produced lower roughness changes than Red Bull^®^ and 360 Energy Drink^®^. This finding suggests that the erosive potential of energy drinks depends not only on pH but also on beverage composition. Red Bull^®^ and 360 Energy Drink^®^ contain higher concentrations of citric acid and sugars, which may enhance erosive activity. Citric acid acts as a chelating agent capable of promoting matrix degradation, filler debonding, and increased surface roughness [10, 11, 15, 24]. In addition, higher sugar content may increase beverage viscosity and prolong acid contact with the composite surface, intensifying degradation.

Time-dependent changes were also observed, as significant differences were detected between baseline and subsequent evaluation periods. This finding supports the cumulative nature of acidic degradation, in agreement with previous studies [11, 12]. Kose et al. reported that energy drinks negatively affect surface roughness, microhardness, and gloss, indicating that even contemporary composites remain susceptible to progressive degradation under acidic conditions [11].

Differences among composites suggest that material composition plays an important role in resistance to acidic exposure. The greater stability of Filtek Z350 XT may be associated with its higher degree of conversion and homogeneous nanofiller distribution, which could reduce surface defects susceptible to acid infiltration [25]. In contrast, Vittra APS and PALFIQUE LX5, which contain more hydrophilic and potentially less stable monomers, showed greater roughness increases over time [19]. Although these properties were not directly evaluated in the present study, they may partially explain the differences observed among the evaluated resin composites.

Loo-Valle et al. also demonstrated that exposure to energy drinks, including Volt^®^, promotes polymer matrix degradation and deterioration of composite surface properties [26]. The acidic components and sugars present in these beverages may favor erosion, plasticization of the resin matrix, and filler particle dislodgement, thereby contributing to increased surface roughness and long-term material deterioration.

To the best of the authors’ knowledge, no previous studies have evaluated the effects of 360 Energy Drink^®^ on dental restorative materials, highlighting the relevance of the present findings. In this study, 360 Energy Drink^®^ produced surface roughness increases comparable to or greater than those observed with Red Bull^®^, particularly in PALFIQUE LX5 and Vittra APS. Although both beverages share similar acidic characteristics, differences in citric acid concentration, sugar composition, and buffering agents may influence their erosive potential. Citric acid promotes chelating activity and matrix degradation, whereas sugars may prolong contact between the beverage and the composite surface. These compositional differences may explain the comparable erosive effects observed for both beverages despite their similar pH ranges.

This study has limitations inherent to its *in vitro* design, which cannot fully reproduce the complexity of the oral environment, including salivary flow, acquired pellicle formation, thermal fluctuations, mechanical wear, and microbiological activity. In addition, complementary surface analyses such as scanning electron microscopy (SEM) or atomic force microscopy (AFM) were not performed, limiting the characterization of microstructural changes induced by acidic exposure. Mechanical properties such as microhardness were also not evaluated, restricting the interpretation of the functional implications of the observed roughness changes. Future studies should incorporate surface, mechanical, and microstructural analyses together with artificial saliva, thermocycling, and toothbrushing simulation to provide a more comprehensive understanding of composite degradation under acidic conditions.

## Data Availability

The datasets are available from the corresponding author upon reasonable request.

## Conclusions

In this *in vitro* study, exposure to energy drinks increased the surface roughness of all evaluated resin composites. The magnitude of degradation depended on both the beverage and the restorative material. Filtek Z350 XT showed greater resistance to roughness changes over time, whereas PALFIQUE LX5 and Vittra APS were more susceptible to acidic exposure, particularly after immersion in Red Bull^®^ and 360 Energy Drink^®^. Although most roughness values remained within clinically acceptable limits, the observed surface alterations suggest that frequent consumption of acidic energy drinks may contribute to the long-term deterioration of composite restorations.
